# The phosphodiesterase 5 inhibitor sildenafil decreases the proinflammatory chemokine IL-8 in diabetic cardiomyopathy: in vivo and in vitro evidence

**DOI:** 10.1007/s40618-018-0977-y

**Published:** 2018-11-10

**Authors:** S. Giannattasio, C. Corinaldesi, M. Colletti, L. Di Luigi, C. Antinozzi, T. Filardi, S. Scolletta, S. Basili, A. Lenzi, S. Morano, C. Crescioli

**Affiliations:** 1 Department of Movement, Human and Health Sciences, Section of Health Sciences, Unit of Endocrinology, Università degli Studi di Roma “Foro Italico”, 00135 Rome, Italy; 2 Institute for Cancer Genetics, University of Columbia, New York, USA; 3 Department of Experimental Medicine, Policlinico Umberto I, Sapienza University of Rome, Rome, Italy; 4 Department of Medical Biotechnologies, University of Siena, Siena, Italy; 5 Department of Internal Medicine and Medical Specialties, Policlinico Umberto I, Sapienza University of Rome, Rome, Italy

**Keywords:** IL-8, Sildenafil, Diabetes, Cardiomyopathy, Inflammation

## Abstract

**Purpose:**

Interleukin (IL)-8 is a proinflammatory C-X-C chemokine involved in inflammation underling cardiac diseases, primary or in comorbid condition, such diabetic cardiomyopathy (DCM). The phosphodiesterase type 5 inhibitor sildenafil can ameliorate cardiac conditions by counteracting inflammation. The study aim is to evaluate the effect of sildenafil on serum IL-8 in DCM subjects vs. placebo, and on IL-8 release in human endothelial cells (Hfaec) and peripheral blood mononuclear cells (PBMC) under inflammatory stimuli.

**Methods:**

IL-8 was quantified: in sera of (30) DCM subjects before (baseline) and after sildenafil (100 mg/day, 3-months) vs. (16) placebo and (15) healthy subjects, by multiplatform array; in supernatants from inflammation-challenged cells after sildenafil (1 µM), by ELISA.

**Results:**

Baseline IL-8 was higher in DCM vs. healthy subjects (149.14 ± 46.89 vs. 16.17 ± 5.38 pg/ml, *p* < 0.01). Sildenafil, not placebo, significantly reduced serum IL-8 (23.7 ± 5.9 pg/ml, *p* < 0.05 vs. baseline). Receiver operating characteristic (ROC) curve for IL-8 was 0.945 (95% confidence interval of 0.772 to 1.0, *p* < 0.01), showing good capacity of discriminating the response in terms of drug-induced IL-8 decrease (sensitivity of 0.93, specificity of 0.90). Sildenafil significantly decreased IL-8 protein release by inflammation-induced Hfaec and PBMC and downregulated IL-8 mRNA in PBMC, without affecting cell number or PDE5 expression.

**Conclusion:**

Sildenafil might be suggested as potential novel pharmacological tool to control DCM progression through IL-8 targeting at systemic and cellular level.

## Introduction

Low-grade inflammation is a critical component of chronic metabolic disorders such as type 2 diabetes (T2D) and is tightly associated with diabetes-induced vascular and cardiac complications [1]. This general inflammatory status, recently referred to as ‘metaflammation’, is common to several comorbid conditions—cardiomyopathy, diabetes, atherosclerosis—and is usually characterized by T helper 1 (Th1) type immune dominance and related biomediators, such as cytokines and chemokines [2]. Several Th1 type biomolecules are engaged in inflammatory processes at systemic, cellular and biomolecular levels, i.e., interleukin (IL)-1, IL-6, IL-8, IL-10, IL-12, tumor necrosis factor (TNF)α, and interferon (IFN)γ. In particular, IL-8 is present since early stages of inflammatory response and remains active for long time [3]; remarkably, this chemokine seems widely involved in cardiac disease, either primary or secondary to dysmetabolism [4]. Indeed, IL-8 serum level is connected with atherosclerosis processes linked to cardiovascular events, in association with baseline cardiovascular risk [5]. Furthermore, higher circulating IL-8 level found in T2D patients vs. non-diabetic subjects was associated with worse inflammatory and cardiometabolic profile [6].

Nowadays, the class of drugs inhibiting phosphodiesterase type 5 (PDE5i), including sildenafil, vardenafil, tadalafil and avanafil, commonly used to treat erectile dysfunction (ED), is documented to exert blunting effects onto Th1-driven processes and biomediators, likely through cGMP/cAMP stabilization [7, 8]. In line with those observations, we have previously reported that sildenafil in diabetic cardiomyopathy (DCM) significantly decreased Th1 type chemokine CXCL10 level, in blood and in human cardiomyocytes [9]. PDE5i seem, indeed, to elicit protective effects in several heart dysfunctions (either primary or secondary to other diseases) such heart failure, ischemia/reperfusion injuries, infarct, ventricular arrhythmias, cardiopulmonary bypass [10–12].

In light of this evidence, it has been hypothesized that PDE5i-elicited cardioprotective effects may rely not only on their undeniable vasoactive action but also on their anti-Th1 type inflammatory processes. Considering the pivotal role of IL-8 during inflammation-induced tissue/cell damage in cardiac diseases, herein we aim to investigate whether sildenafil can target IL-8 level in T2D subjects with DCM and in human endothelial and peripheral immune cells under maximal Th1 inflammatory challenge. To this purpose, we measured IL-8 in sera from T2D patients at the onset of cardiomyopathy secondary to diabetes, before and after chronic treatment with sildenafil (100 mg/day for 3 months) vs. placebo. Baseline chemokine levels were measured in sex- and age-matched healthy subjects. In addition, we investigated the effect of sildenafil onto IL-8 released by human endothelial and immune cells activated by IFNγ+TNFα or phytohaemagglutinin (PHA), respectively.

## Materials and methods

### Chemicals

Plastic for cell cultures and disposable filtration units were purchase from Corning (Milan, Italy). Dulbecco-modified eagle medium (DMEM)/Ham’s F-12 medium (ratio 1:1) with and without phenol red, RPMI-1640 Medium, phosphate-buffered saline Ca^2+^/Mg^2+^-free (PBS), bovine serum albumin (BSA) fraction V, antibiotics, EDTA-trypsin solution, Bradford reagent were from Sigma-Aldrich Corp. (St. Louis, MO, USA). Fetal bovine serum (FBS) was from Hyclone (Logan, UT, USA). Recombinant human interferon (IFN-γ) and recombinant human Tumor Necrosis Factor alpha (TNFα) were from Peprotech^®^ (RockyHill, NJ, USA). For RNA extraction, TRIzol RNA isolation reagent was purchased by Ambion™; for reverse transcription 10 mM dNTP mix, random primers, RNaseOUT™ Ribonuclease inhibitor and SuperScript^®^ III Reverse were purchased from Invitrogen. SYBR^®^ Green PCR Master Mix for qPCR was from Life Technologies™ (Applied Biosystems^®^). All reagents for SDS-PAGE were from Millipore (Billerica, MA, USA). l-Glutamine was from Gibco Laboratories (Grand Island, NY). Polyclonal rabbit anti-PDE5 and monoclonal mouse anti-β-actin were from Santa Cruz (CA, USA).

### Subjects

Frozen samples from 46 subjects with DCM were analyzed (Clinical Trial Registration—URL: http://www.clinicaltrials.gov. Unique identifier: NCT00692237) [13]. The protocol was approved by Hospital Ethics Committee Policlinico Umberto I—Sapienza University Hospital of Rome. This was a randomized controlled trial with patients allocated to receive 100 mg/day sildenafil for 3 months (30 subjects) or placebo (16 subjects). Eligible men with T2D were recruited from the outpatient of Policlinico Umberto I—Sapienza University Hospital of Rome, the inclusion criteria were: T2D > 1 year; normal blood pressure (BP) or treated hypertension with achievement of a target of  ≤ 130/80 mmHg; glycated hemoglobin (HbA1c) < 10%; body mass index (BMI) < 40; Table 1 summarizes patient characteristics. The exclusion criteria were: prior or current use of PDE5i; use of exogenous insulin, thiazolidinediones, or spironolactone; substance abuse; history of cardiovascular disease, proliferative retinopathy, autonomic neuropathy; symptoms or signs of ischemic heart disease during cardiac evaluations at enrollment; contraindications to sildenafil use or cardiac magnetic resonance (CMR) imaging. Concomitant medications (anti-hypertensives, statins, etc.) were not changed between the months prior to the study and 1 month after it has finished. All blood samples were collected from peripheral vein and serum was obtained by centrifugation (3000 rpm for 10 min at 4 °C); aliquots were stored at −80 °C until analyzed.

**Table 1:** Clinical characteristics of the study populations

Variable	Diabetic cardiomyopathy sildenafil arm	Diabetic cardiomyopathy placebo arm
Numbers	30	16
Age (years)	61.4 ± 1.4	60.7 ± 1.3
BMI (kg/m^2^)	28.4 ± 0.9	27.6 ± 0.8
Glycemia (mmol/L)	8.4 ± 1.9	8.2 ± 1.6
HOMA-index	6.3 ± 0.8	7.5 ± 0.8
HbAlc (%)	7.9 ± 0.2	7.3 ± 0.3
Total cholesterol (mmol/L)	4.8 ± 1.0	4.4 ± 0.2
HDL cholesterol (mmol/L)	1.05 ± 0.2	1.2 ± 0.06
LDL cholesterol (mmol/L)	3.01 ± 0.98	2.43 ± 0.20
Triglycerides (mmol/L)	1.4 ± 0.7	1.4 ± 1.1
Mean systolic BP (mm Hg)	136.1 ± 2.2	131.4 ± 2.8
Mean diastolic BP (mm Hg)	79.7 ± 1.6	78.9 ± 1.4
LVMi (g/m^2^)	124.6 ± 4.7	114.1 ± 5.9
EDVi (mL/m^2^)	61.4 ± 1.7	60.76 ± 2.3
Ejection Fraction (%)	61.6 ± 1.5	59.1 ± 1.90
BNP (pmol/L)	2.6 ± 0.3	3.02 ± 1.0
NT proBNP (pg/ml)	70 ± 11.9	72.2 ± 18.5
Serum IL-8 levels (pg/ml)	23.7 ± 6	130.33 ± 89.7

Values of the different clinical parameters did not differ between sildenafil and placebo arm

*BMI* body mass index, *HOMAi* Homeostasis model assessment index, *HbA1c* Hemoglobin A1c, *HDL* High-density lipoprotein, *LDL* Low-density lipoprotein, *BP* Blood pressure, *LVMi* Left ventricular mass index, *EDVi* End-diastolic volume index, *BNP* brain natriuretic peptide, *NT proBNP* N-terminal pro b-type natriuretic peptide

One additional group of subjects, matched for sex and age, were analyzed for comparisons: 15 subjects without any pathology (healthy subjects). Written informed consent was collected for all subjects.

### Cell cultures

Human fetal aortic endothelial cells (Hfaec) were obtained from aortic ascendant tracts collected after voluntary abortion (10–12 weeks of gestation) characterized and maintained as described elsewhere [14]. Legal abortions were performed in authorized hospitals; certificates of consent were obtained. The use of human fetal tissue for research purposes conforms with the principles outlined in the Declaration of Helsinki and was approved by the committee for investigation in humans of the Azienda Ospedaliero-Universitaria Careggi, Florence, Italy (protocol no. 6783–04).

Peripheral blood mononuclear cells (PBMCs) were isolated from buffy coats obtained from healthy adult anonymous donors in accordance with local ethical committee; approval by Azienda Policlinico Umberto I Rome Italy, accordance with the principles outlines in the Declaration of Helsinki; written consents were obtained. Heparinized blood, collected from peripheral vein, was centrifuged on Ficoll–Hystopaque gradient following manufacture’s protocol. PBMCs were cultured in RPMI supplemented with 10% FBS, 2 mmol/l l-glutamine and antibiotics.

### IL-8 secretion assays

#### Serum determination

Human serum levels of IL-8 were measured using a magnetic bead-based multiplex assay (Bio-Plex Pro™ Human Cytokine, Chemokine and Growth factor assay, Bio-Rad laboratories, Inc.) according to the manufacturer’s protocol. A broad sensitivity range of standards (between 1.95 and 95.000 pg/ml; Bio-Rad Laboratories, Inc.) was used to enable the quantification of a dynamic wide range of cytokine concentrations and provide the greatest sensitivity. Data acquisition was performed by Bio-Plex 200 System™ (Bio-Rad Laboratories, Inc.) which uses Luminex fluorescent-bead-based technology (Luminex) with a flow-based dual laser detector with real-time digital signal processing to facilitate the analysis of up to 100 different families of color-coded polystyrene beads and allow multiple measurements of the sample ensuing in the effective quantification of cytokines. Data analysis was performed by Bio-Plex Manager™ 6.0 software (Bio-Rad Laboratories, Inc.). Serum samples were run in triplicate at least twice. Data were expressed as serum IL-8 concentration divided in “healthy” (subjects with no pathology), “baseline” (subjects with DCM before any therapy) and “+ sildenafil” and “+ placebo” (subjects with DCM and with sildenafil or placebo therapy after 3 months).

#### Cell supernatant determination

Hfaec (4000 cells/well) were seeded in 96-well flat bottom plates, maintained in phenol red- and serum-free medium overnight and incubated in serum-free medium containing 0.1% BSA with sildenafil (1 µM) for 24–48 h; cells in serum-free medium containing 0.1% BSA and vehicle were used as control.

PBMC (150,000 cells/well) were seeded in 96-well round bottom plates in their growth medium and stimulated with PHA (2%) with or without sildenafil (1 µM) for 24 h and 48 h. Cells in growth medium with vehicle and PHA were used as controls. Sildenafil was added each day in each cell type.

IL-8 levels were measured in cell cultures supernatants using commercially ELISA (enzyme-linked immunosorbent assay) available kits, according to the manufacturer’s recommendations (R&D Systems—Minneapolis). The sensitivity ranged was from 1.5 to 7.5 pg/ml for IL-8. The intra- and inter-assay coefficients of variation were 4.6 and 8.1%. Quality control pools of low, normal, and high concentrations for all parameters were included in each assay. Samples were assayed in triplicate or quadruplicate. Protein measurement to normalize the concentration of secreted cytokines was performed as reported elsewhere [15]. Experiments were performed four times with different cell preparations. Data were expressed as percent of inhibition, calculated on IFNγ + TNFα- and/or PHA release, taken as 100%.

### RNA extraction, reverse transcription and real-time quantitative PCR

35,000 Hfaec were seeded in 35-mm culture dishes and maintained for 24 h in their growth medium; after 12 h starvation (medium without serum and without phenol red), cells were stimulated for 24 h with a combination of IFNγ (1000 U/ml) + TNFα (10 ng/ml) with or without sildenafil (1 μM), in serum-free medium with 0.1% BSA, cells in serum-free medium containing 0.1% BSA and vehicle were used as control; 200,000 PBMC were seeded onto 96-well round bottom plates in their growth medium and stimulated for 48 h with 2% PHA with or without sildenafil (1 μM), cells in growth medium and vehicle were used as control. Total RNA was extracted from cultured cells using TRIzol^®^ RNA Isolation Reagents (Ambion™) according to the manufacturer’s instructions and described also elsewhere [16]. Single-stranded cDNA was obtained by reverse transcription of 1 μg of total RNA. RT-qPCRs were performed using 7500 Real Time System (Applied Biosystems^®^) with SYBR-green fluorophore; 40 ng of cDNA were used as template and cycling parameters were 95 °C for 10 min, followed by 40 cycles of 15 s at 95 °C, 1 min at 60 °C, 30 s at 95 °C, 15 s at 60 °C. Fluorescence intensities were analyzed using the manufacturer’s software (7500 Software v2.05) and relative amounts were obtained using the 2 − ∆∆Ct method and normalized for the ß-actin. Data are expressed as percent of inhibition of IFNγ + TNFα- or PHA-induced IL-8 gene expression, taken as 100%. Primers for IL-8 were: forward (TCCTGATTTCTGCAGCTCTGTG) and reverse (GTCCAGCAGAGCTCTCTTCCAT); for β-actin, forward (CTGAACCCCAAGGCCAAC) and reverse (AGCCTGGATAGCAACGTACA).

### Cell proliferation

For cell proliferation assay, Hfaec (4000 cells/well) and PBMC (150,000 cells/well) were seeded in 96-well flat and round bottom plates, respectively, as reported in cell supernatant determination subparagraph.

In each cell type, sildenafil was added each day. Cell number counting was assayed by hemocytometer as previously reported [17].

### Western blot analysis

Hfaec and PBMC were seeded and maintained for western blot analysis, until they reach sufficient number to lyse in RIPA Buffer. Protein concentration measurement was performed with Bradford Reagent. Protein aliquots (20 μg) were processed, loaded in 10% SDS-PAGE gel, transferred on nitrocellulose membranes, and incubated with primary Abs appropriately diluted in Tween Tris-buffered saline (TTBS; for anti-PDE5 1:500), followed by peroxidase-conjugated secondary IgG (1:10,000). Proteins were revealed by the enhanced chemiluminescence system (ECL plus; Millipore). Image acquisition was performed with Image Quant Las 4000 software (GE Healthcare). Western blot analysis was performed for three experiments with different cell preparations.

### cGMP quantification

cGMP determination has been performed onto 10^6^ cells after overnight starvation and 30 min incubation with or without sildenafil, using a commercially available kit (cGMP ELISA Kit from Enzo Life Sciences; Vinci, Fi, Italy) with 3-isobutyl-1-methylxanthine (IBMX) as internal positive assay control.

### Statistical analysis

Our research was conceived as a “proof of concept” study. The statistical analysis was performed using 12.0 SPSS (SPSS Inc, Chicago, IL, USA). The Kolmogorov–Smirnov test was used to test for normal distribution of the data. Continuous data were compared using unpaired Student’s *t* test or Mann–Whitney test when request. Receiver operating characteristic (ROC) curve was constructed to assess the capacity of discriminating the response in terms of sildenafil-induced IL-8 decrease. Briefly, ROC curve gives a graphic representation of the relationship between true-positive fraction (sensitivity) and false-positive fraction (1-specificity). ROC curve can be assessed by plotting the values of 1-Sp against Se in a squared box, where the ROC’s area under the curve (AUC) is used to measure the performance of a diagnostic test. The AUC range is in the interval 0.5–1.0, so that the greater the area, the better the performance of the variable being examined. Sigmoid curves were performed using GraphPad Prism 5 software (GraphPad Software, Inc., La Jolla, CA, USA) and SPSS 12.0 software package. A P value less than 0.05 was considered significant and corrected for comparison using the Dunnett’s or Bonferroni’s post hoc test, where appropriate. Data were expressed as mean ± SE.

## Results

### IL-8 levels in healthy subjects and DCM patients before and after sildenafil or placebo intake

To verify whether PDE5i could affect serum IL-8, we measured the chemokine blood level in DCM subjects before (baseline) and after sildenafil or placebo intake vs. healthy subjects.

**Fig. 1:**
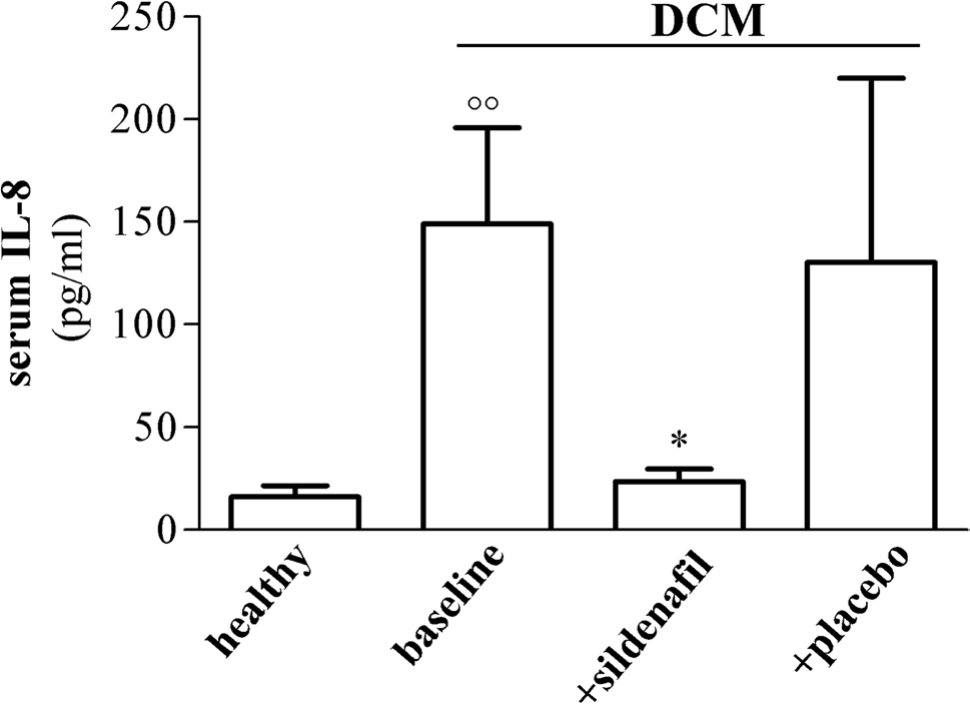
Serum IL-8 levels in DCM patients before (baseline) and after 3 months sildenafil or placebo, vs. healthy subjects. DCM patients showed higher baseline levels of IL-8 vs. healthy subjects (149.14 ± 46.89 pg/ml vs. 16.17 ± 5.38 pg/ml); IL-8 was significantly reduced (23.7 ± 6 pg/ml) close to healthy levels in DCM patients after sildenafil. Conversely, serum IL-8 remained high after placebo (130.33 ± 89.74 pg/ml). *p < 0.05 vs. baseline; °°*p* < 0.01 vs. healthy. Results (mean ± SE) are expressed as serum IL-8 (pg/ml)

In DCM patients, baseline IL-8 levels were about tenfold higher vs. healthy subjects (149.14 ± 46.89 pg/ml vs. 16.17 ± 5.38 pg/ml, *p* < 0.01) (Fig. 1). Sildenafil after 3 months decreased IL-8 close to similar values as found in healthy subjects (23.7 ± 6 pg/ml, *p* < 0.05 vs. baseline), whereas placebo left essentially unvaried baseline IL-8 in blood (130.33 ± 89.74 pg/ml) of DCM patients.

### Assessment of IL-8 serum response to sildenafil in DCM patients

**Fig. 2:**
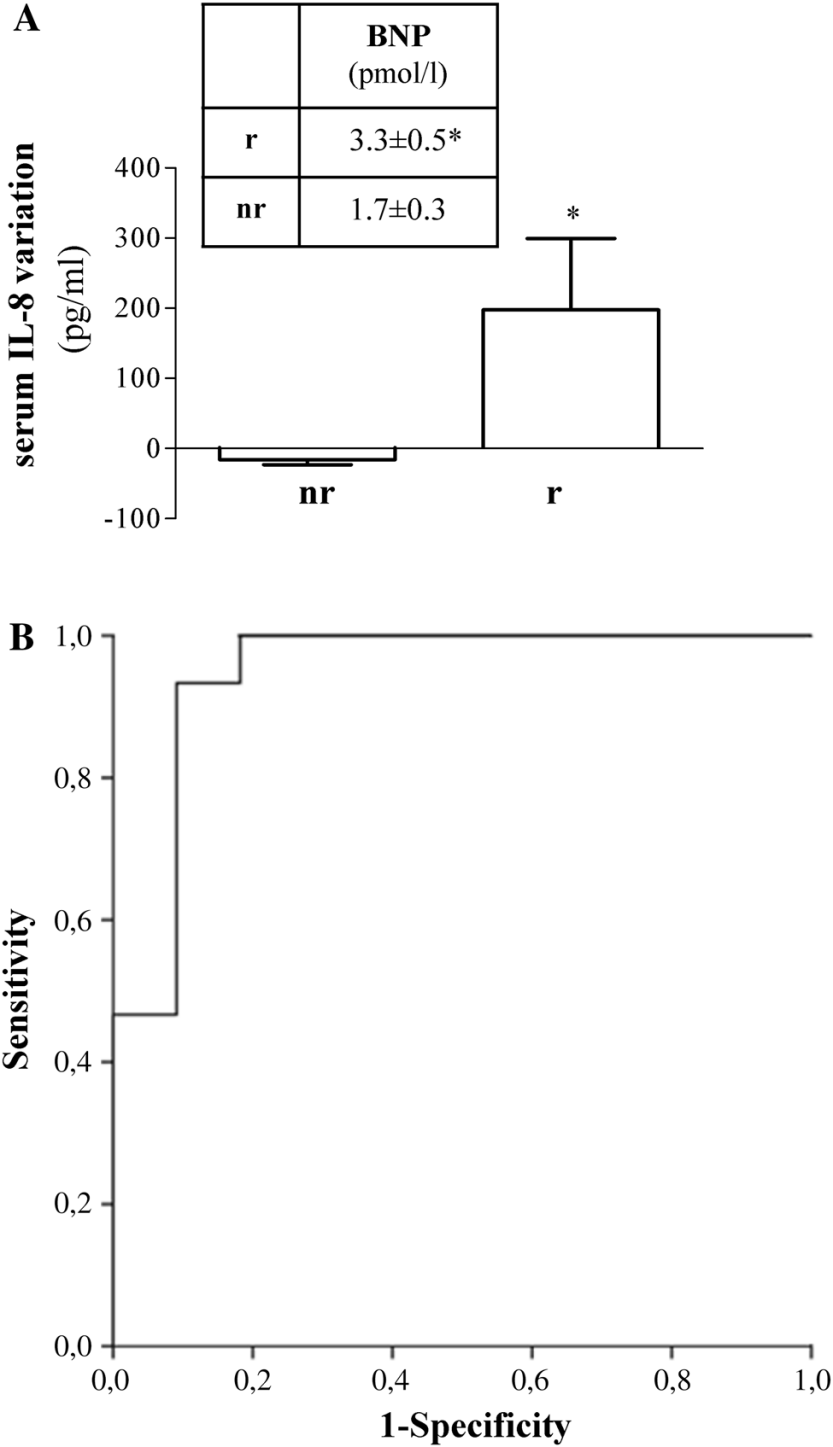
Effect of sildenafil on circulating IL-8 in subjects with DCM. **a** IL-8 serum level categorized DCM patients as responders (*r*) and non-responders (*nr*) to sildenafil; positive 197.55 ± 101.8 pg/ml) or null/negative (− 16.05 ± 6.97 pg/ml) variation was observed after sildenafil intake; *p < 0.05 * r*vs. *nr.* Inset: Baseline BNP was significantly higher in* r* vs.* nr*, *p < 0.05. Data of IL-8 (mean ± SE) are expressed as serum variation vs. baseline level (pg/ml); BNP (mean ± SE) was expressed as pmol/l. **b**. Receiver operating characteristic (ROC) curve. The area under the ROC curve is 0.945 (95% confidence interval of 0.772 to 1.0, *p* < 0.01), with a sensitivity of 0.93 and a specificity of 0.90

To further estimate the effect of sildenafil in DCM patients, we analyzed IL-8 serum level constructing ROC curve based on the response to sildenafil. Responders (*r*) and non-responders (*nr*) to sildenafil were defined as those patients who had positive or null/negative response to the drug, respectively. *R* and nr had IL-8 baseline value of 197.55 ± 101.8 pg/ml and − 16.05 ± 6.97 pg/ml, respectively (Fig. 2a). Out of all clinical parameters analyzed, B-type natriuretic peptide (BNP), used as clinical biomarker for congestive heart failure diagnosis, was significantly higher in r vs. nr (*p* < 0.05, inset of Fig. 2a), albeit within normal range.

Receiver operating characteristic (ROC) curve for IL-8 was 0.945 (95% confidence interval of 0.772 to 1.0, *p* < 0.01), showing good capacity of discriminating the response in terms of drug-induced IL-8 decrease (sensitivity of 0.93, specificity of 0.90) (Fig. 2b). ROC analysis identified a low IL-8 cut-off value (12.25 pg/ml).

### Effect of sildenafil onto IL-8 protein secretion and gene expression in Hfaec and PBMC

**Fig. 3:**
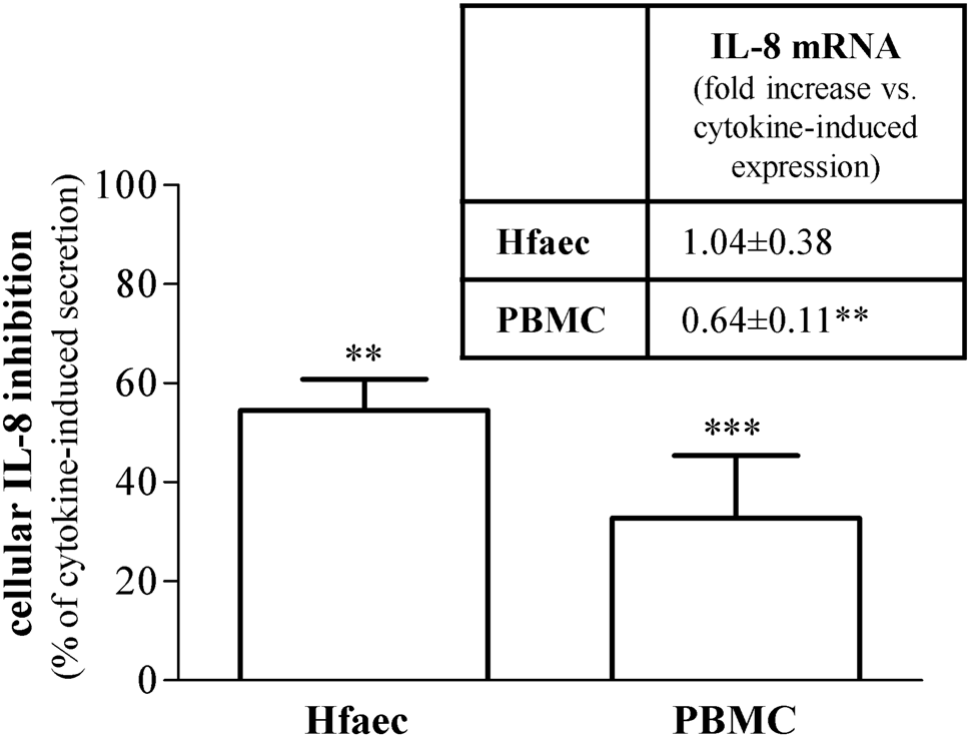
Effect of sildenafil on IL-8 release induced by inflammatory stimuli in Hfaec and PBMC. IL-8 protein release was significantly counteracted by sildenafil in Hfaec and PBMC (percent of inhibition 54.54% ± 1.09 and 32.80% ± 0.55); results (mean ± SE) are expressed as percent of inhibition, calculated on IFNγ + TNFα- or PHA-induced IL-8 maximal secretion, taken as 100%; ***p < 0.001, **p < 0.01. Inset: IL-8 mRNA expression was reduced only in PBMC treated with sildenafil. mRNA levels—relative to β-actin mRNA, used as endogenous control—are expressed as (mean ± SE) percent of inflammatory stimuli-induced IL-8 mRNA expression, taken as 1; **p < 0.01. Data are obtained from three to seven experiments in triplicate, using different cell preparations

To verify whether PDE5 inhibition might affect Th1-driven IL-8 at cellular level, we investigated the effect of sildenafil (1 µM) onto chemokine protein secretion and gene expression in Hfaec and in PBMC after maximal inflammatory challenge (with IFNγ + TNFα or PHA, respectively) (Fig. 3). Drug concentration was chosen based on the near therapy dose, according to pharmacokinetics (Cmax and area under the time–concentration curve).

Sildenafil significantly inhibited IL-8 protein release by over 50% in Hfaec and 30% in PBMC (*p* < 0.01 and *p* < 0.001 vs. inflammatory stimuli-induced secretion, taken as 100%). IL-8 mRNA expression was also significantly reduced by sildenafil in PBMC (*p* < 0.01 vs. PHA-induced IL-8 gene expression, taken as 1), whereas it was unaffected in Hfaec (inset of Fig. 3).

### cGMP measurement and PDE5 expression before and after sildenafil in Hfaec and PBMC

**Fig. 4:**
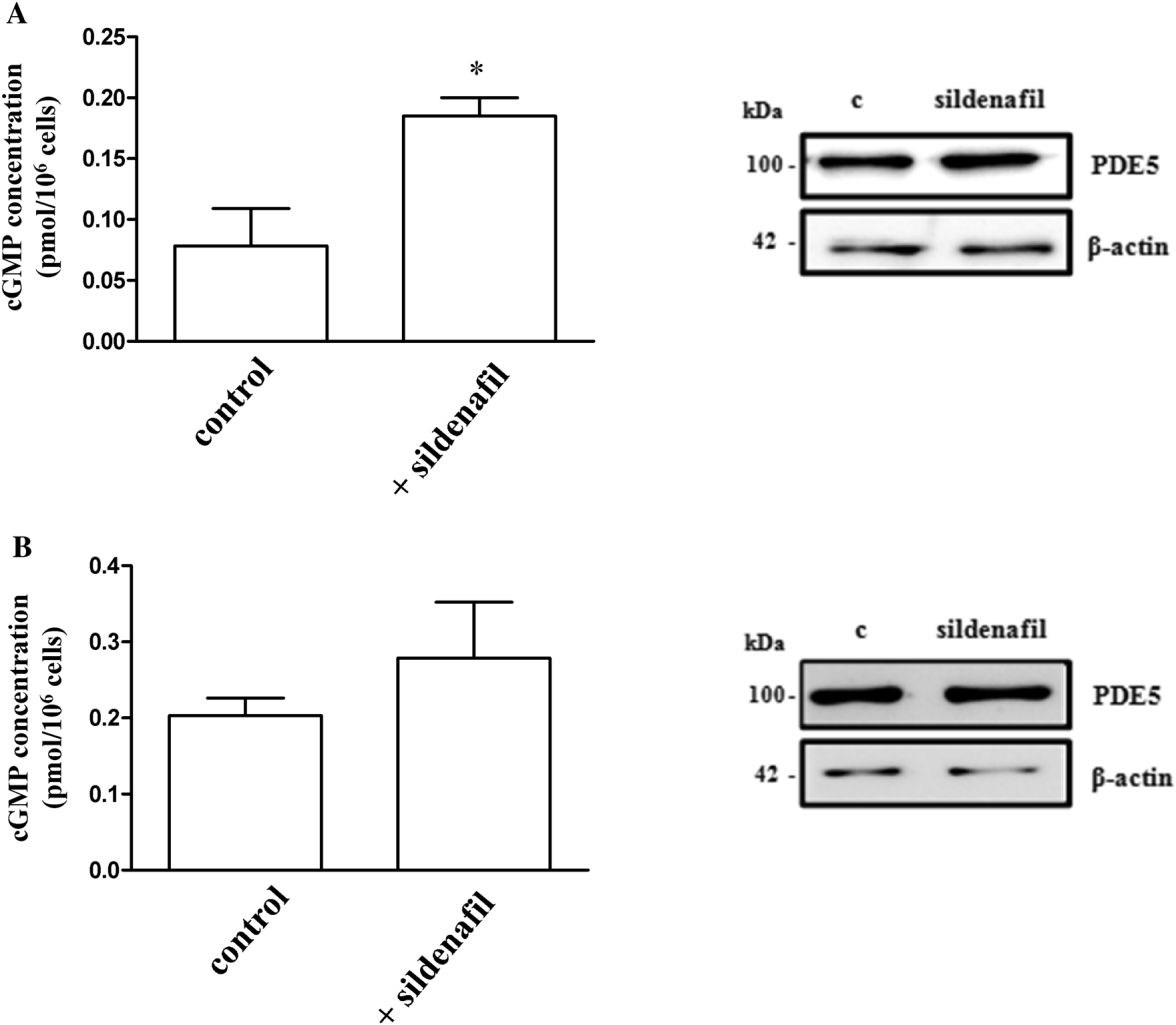
Effect of sildenafil on cGMP stabilization and PDE5 expression. **a** cGMP concentration was significantly higher in Hfaec treated with sildenafil (1 µM, 30 min); *p < 0.05 vs. control cells. *Inset* Western blot analysis showed no changes of PDE5 protein expression in Hfaec before and after sildenafil; β-actin was used as loading control. **b** cGMP amount in PBMC after 30 min of sildenafil (1 µM) showed a trend to increase though not statistically different vs. control. *Inset* Western blot analysis showed no changes in PDE5 protein expression before and after sildenafil; β-actin was is the loading control. Results in panel A (mean ± SE) are expressed as cGMP concentration (pmol/10^6^ cells) and derived from two different preparations for each cell type; the blots in panel **a** and **b** are representative of data deriving from two/three different preparations of both cell types

Since sildenafil is a selective cGMP-dependent inhibitor of PDE5 activity, we measured cGMP amount and PDE5 expression in human endothelial and immune cells before and after sildenafil (Fig. 4).

Sildenafil (1 µM) significantly increased cGMP level after 30 min in Hfaec (*p* < 0.05 vs. control; Fig. 4a); cGMP in PBMC showed the same trend to rise after sildenafil, although not reaching statistical significance, like due to the high number variability linked to data deriving from isolated PBMC (Fig. 4b). PDE5 protein expression neither changed in Hfaec (inset of Fig. 4a) nor in PBMC (inset of Fig. 4b) after exposure to sildenafil (1 µM) for 24 h.

### Effect of sildenafil on Hfaec and PBMC cell number

**Fig. 5:**
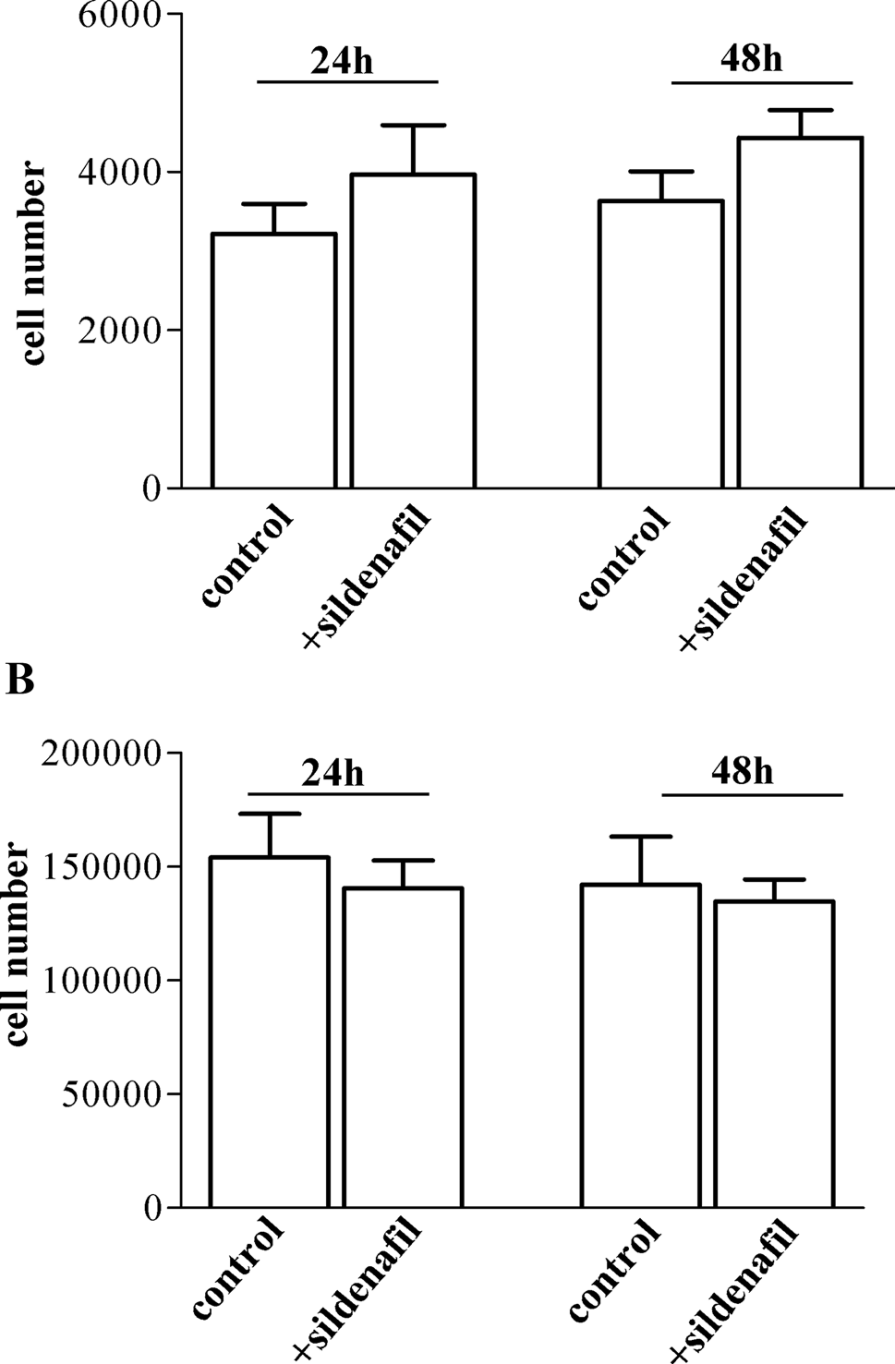
Effect of sildenafil on human cardiac, endothelial and immune cell viability. **a** Hfaec cell number did not change after sildenafil (1 µM) for 24 h or 48 h vs. control (without drug). **b** PBMC cell number was not modified by sildenafil (1 µM) after 24 h and 48 h vs. control cells. Results derived from at least four different cell preparations for each cell type and are expressed as (mean ± SE)

To exclude that sildenafil could affect human endothelial or immune cell number, we performed proliferation tests and found no significant effect in Hfaec and PBMC after 24–48 h exposure to sildenafil (1 µM) as compared with control cells (Fig. 5a, b).

## Discussion

The main finding of the study is that sildenafil, not placebo, significantly reduced the higher IL-8 serum concentration associated with DCM to similar levels as in healthy subjects. ROC curve for IL-8 showed good capacity to discriminate the response in terms of sildenafil-induced serum IL-8 decrease. Furthermore, sildenafil inhibited IL-8 protein release by inflammation-activated human immune and endothelial cells, likely with transcriptional and post-transcriptional regulation.

The research onto inflammatory diseases has rapidly progressed in the last 50 years leading researchers and physicians to redefine many diseases as “inflammatory” disorders, including metabolic and heart diseases [18, 19]. The concept of metaflammation as the first trigger common to several pathologies has come from growing evidence on the association between chronic inflammatory diseases and elevated blood levels of inflammatory markers [20, 21]. Th1 type chemokines regulate several inflammatory mechanisms considered the signature of metaflammation, essentially orchestrating immune cell recruitment and complex inter- and intracellular signaling. Among those biomediators, IL-8 or CXCL8, a prototypical chemoattractant component of ELR + C-X-C chemokine subfamily, derives from a variety of tissue and blood cells, and it is responsible for and involved in a wide range of inflammatory processes determining vascular bed homeostasis unbalance, with consequence on endothelial mediator release and vascular tone [22]. Remarkably, each cellular component in the vascular wall has been identified as a potential source of IL-8 significantly contributing to the inflammatory micro-environment maintenance of the injured vascular bed [3]. Differently from many other proinflammatory molecules, typically made and cleared in vivo within hours, IL-8 is present early and remains active for a prolonged time [3] during inflammatory response. IL-8 retains distinct target specificity for neutrophils which, once recruited, release granule enzymes degrading connective tissue constituents and determining intra- and extracellular injurious re-arrangements [23]. Transendothelial cell migration in chronic setting induced by IL-8 underlies endothelial dysfunction, as reported in several vascular diseases, from atherosclerosis, to aortic aneurysm formation and hypertension [24].

Thus far, since endothelial instability underlies cardiac disease in the presence or absence of comorbidity [25, 26], the ability of sildenafil to counteract vascular inflammation by targeting IL-8 could potentially be of clinical interest. Previous data showed that daily administration of sildenafil reduced vascular inflammation and improved endothelial function in T2D patients [27]. Clinical evidence sustains a strong association between PDE5i use and reduced mortality rate and hospitalization in a cohort of T2D patients with attendant high cardiovascular risk [28]. PDE5 inhibition seems also to exert favorable effects by enhancing enzymatic antioxidant system capacity [29]. Of note, those effects were not achieved with other class of vasoactive drugs as prostaglandins.

Concerning IL-8 cut-off low level (12.25 pg/ml), we observed that it was below the mean IL-8 concentration found in healthy subjects. Thus, we only can speculate that DCM patients would get benefit from sildenafil in terms of chemokine decrease when their baseline IL-8 value is much higher than that cut-off level.

Of note, the majority of DCM subjects classified as r to sildenafil concerning serum IL-8 level was previously grouped as r to the drug in terms of serum CXCL10, a Th1 type ERL chemokine [9], and also retained higher baseline BNP, although still within normal reference range.

We speculate that DCM subjects showing from the beginning higher Th1-type inflammatory chemokines might be perhaps more prone to progress in cardiomyopathy.

Moreover, sildenafil significantly counteracted IL-8 protein release from endothelial and immune cells—both known sources of the chemokine contributing to amplify inflammation—likely through post-transcriptional mechanism(s) in Hfaec, and transcriptional regulation in PBMC.

While in Hfaec sildenafil effect is undeniably mediated by cGMP stabilization as reflected by the significant increase, cGMP rise in sildenafil-treated PBMC, albeit showing a trend to increase, did not reach statistical significance, likely due to high number variability. PDE5 protein expression was not modified by sildenafil in either cell type in line with the previous literature [30]. However, detectable increase in PDE5 protein expression is reported after longer times (up to 7-days incubation) and higher dosage (25 µM) [31, 32].

In conclusion, from the present and previous data, sildenafil is able to target high level of ERL +/ERL–C-X-C chemokines, as IL-8 and CXCL10, both known to play pivotal roles in initiation and progression of micro-environmental immune/inflammatory processes leading to self-detrimental perpetuating loops established between local and systemic areas [33–35].

We would like to point out the potential clinical impact of C-X-C chemokine targeting, since those biomolecules act as immune/inflammatory triggers starting from an early temporal frame, likely preceding clinical cardiac sign manifestation. Indeed, while sildenafil modified metabolic parameters such as Hemoglobin A1c, post-prandial glycemia and lipidemic profile [36], it failed to modify standard markers of chronic cardiac decompensation, such as ejection fraction, mass and volume index or blood pressure. We speculate on this (lacking) effect considering that all DCM patients enrolled were at the initial stage of cardiac disease, with no clinical signs of ischemia and left ventricular (LV) function well preserved.

Accordingly, it has been shown that T2D man with ED still asymptomatic for cardiovascular disease showed higher monocyte oxidative activity and endothelial dysfunction [37], both additionally reduced with sildenafil given in combination with l-carnitine [38].

Likely, medium/long term follow-up would reveal whether sildenafil-induced IL-8 inhibition in blood could possibly relate with variation of some clinical parameters associated with patient status.

In addition, further medium/long-term investigations likely will elucidate whether the lack of any modification of circulating IL-8 after 4 weeks of sildenafil in T2D patients, as previously reported [39] could be possibly explained with differences in treatment, including time-frame and protocols.

So far, our results support the hypothesis that sildenafil could be a good candidate as a therapeutic option (may be as sparing-agent?) in patients with metabolic disorders, including comorbid conditions, at increased risk of cardiovascular abnormalities, often ameliorated by testosterone replacement [40, 41]. Furthermore, sildenafil retains a better cardiovascular profile vs. other PDE5i, as demonstrated by well-established overall safety after its use, with the limitation to avoid co-administration with long- and short-acting nitrate preparations [42]. More recently, it has been reported in animals that PDE5 inhibition by sildenafil, not tadalafil, decreased edema in lung ischemia–reperfusion injury during cardiopulmonary blocks [43].

Our investigation has several limits besides the small sample size analyzed, first the lack of subjects affected by T2D or cardiomyopathy alone with and without sildenafil treatment. Moreover, considering the high number of biomediators engaged in metaflammation during comorbid conditions, it is mandatory to analyze a combination/ratio of biomediators. As an example, when evaluating vascular homeostasis and inflammation in diabetes, it is mandatory to consider molecules such as angiopoietins, critically involved in those processes [44]. Indeed, integrated analysis including an amplified number of noninvasive biomarkers aimed to obtain implemented algorithms has been recognized since a while to retain better predictive values, useful for validated decision models in several diseases [45, 46]. Due to their positive impact on both patients health care and efficient use of resources, standard risk algorithms incorporating different type of biomarkers, i.e., including sex/gender-related variables, are nowadays strongly encouraged also in T2D and its complications [47–49]. In this direction, we are planning our future investigations. Finally, the observed cut-off value of IL-8 resulted very low and below the mean IL-8 concentration found in healthy subjects. Hence, we believe that our findings are to be mitigated and taken cautiously, as they need to be confirmed in larger sample size studies.

Nevertheless, previous and present data suggest that sildenafil could be recommended with the novel indication to control metabolic status and early stage inflammatory processes underlying DCM and warrant further in vivo and in vitro studies on PDE5i-promoted cardioprotection to assess the related fine-tuned biomolecular mechanisms.
